# Influence of a novel scaffold composed of polyurethane, hydroxyapatite, and decellularized bone particles on the healing of fourth metacarpal defects in mares

**DOI:** 10.1111/vsu.13608

**Published:** 2021-05-05

**Authors:** Remigiusz M. Grzeskowiak, Karrer M. Alghazali, Silke Hecht, Robert L. Donnell, Thomas J. Doherty, Christopher K. Smith, David E. Anderson, Alexandru S. Biris, Henry S. Adair

**Affiliations:** ^1^ Department of Large Animal Clinical Sciences The University of Tennessee College of Veterinary Medicine Knoxville Tennessee USA; ^2^ Center for Integrative Nanotechnology Sciences University of Arkansas at Little Rock Little Rock Arkansas USA; ^3^ Department of Small Animal Clinical Sciences The University of Tennessee College of Veterinary Medicine Knoxville Tennessee USA; ^4^ Department of Biomedical and Diagnostic Sciences The University of Tennessee College of Veterinary Medicine Knoxville Tennessee USA

## Abstract

**Objective:**

To determine the effect of a novel scaffold, designed for use in bone regeneration, on healing of splint bone segmental defects in mares.

**Study design:**

In vivo experimental study.

**Sample population:**

Five adult mares (4–10 years old; mean weight, 437.7 kg ± 29 kg).

**Methods:**

Bilateral 2‐cm full‐thickness defects were created in the fourth metacarpal bones (MCIV) of each horse. Each defect was randomly assigned to either a novel scaffold treatment (n = 5) or an untreated control (n = 5). The scaffold was composed of polyurethane, hydroxyapatite, and decellularized bone particles. Bone healing was assessed for a period of 60 days by thermography, ultrasonography, radiography, and computed tomography (CT). Biopsies of each defect were performed 60 days after surgery for histological evaluation.

**Results:**

On the basis of radiographic analysis, scaffold‐treated defects had greater filling (67.42% ± 26.7%) compared with untreated defects (35.88% ± 32.7%; *P* = .006). After 60 days, CT revealed that the density of the defects treated with the scaffolds (807.80 ± 129.6 Hounsfield units [HU]) was greater than density of the untreated defects (464.80 ± 81.3 HU; *P* = .004). Evaluation of histology slides provided evidence of bone formation within an average of 9.43% ± 3.7% of the cross‐sectional area of scaffolds in contrast to unfilled defects in which connective tissue was predominant throughout the biopsy specimens.

**Conclusion:**

The novel scaffold was biocompatible and supported bone formation within the MCIV segmental defects.

**Clinical significance:**

This novel scaffold offers an effective option for filling bone voids in horses when support of bone healing is indicated.

**Abbreviations**
3D: three‐dimensional
CT: computed tomography
DBP: decellularized bone particles
HA: hydroxyapatite
HU: Hounsfield units
MCIV: fourth metacarpal bone
PACS: picture archival and communication system
PU: polyurethane
ROI: region of interest

## 1 INTRODUCTION

Injuries resulting in bone defects and reduction of bone structural support are common in equine athletes. Fractures have been estimated to cause up to 63% of fatal injuries in the racing industry,^1^, ^2^, ^3^ and approximately 34% of fractures are comminuted with variable degrees of bone loss.^2^, ^4^ Fracture healing remains challenging in horses due delayed union, nonunion, fracture repair failures resulting from high‐stress loads, and infections.^5^, ^6^, ^7^, ^8^ Furthermore, prolonged convalescence is associated with life‐threatening complications such as contralateral limb laminitis or colitis.^9^, ^10^

Autologous bone grafts are the clinical standard to fill voids in bone because of the low rejection risk as well as the ability to transfer viable osteoprogenitor cells.^11^, ^12^ Limitations of autografts include the requirement of a second surgery site, the risk of infection of the second surgery site, and a limited amount of donor tissue available.^13^, ^14^ Bone fillers should be osteoinductive, osteoconductive, and osseointegrative.^15^ Synthetic biomedical implants for use as bone or cartilage fillers in tissue defects have been reported as potential options in horses.^16^, ^17^, ^18^, ^19^ Limitations of currently available bone fillers include loose granular materials that are subject to migration or dispersion in adjacent tissues, lack of durable and malleable three‐dimensional (3D) structures, limited ability to adjust devices to fit irregular bone voids, inconsistent porosity, and variable implant degradation rates that may impede bone formation.^20^

Recently, a novel scaffold was invented for use as a bone filler to aid in the healing of bone defects and voids. The scaffold is a porous, 3D block structure composed of polyurethane (PU), hydroxyapatite (HA), and decellularized bone particles (DBP).^21^, ^22^, ^23^ The PU used in this platform is degradable and biocompatible and has malleable properties, ensuring preservation of the integrity of the structure in vivo.^21^, ^22^ Implanted scaffolds are designed to degrade over time and to be gradually replaced by regenerating bone tissue. Nanostructured HA has been shown to promote osteoblastic cell adhesion and proliferation, provide calcium‐containing minerals, and change the surface energy of coated scaffolds to control initial protein adsorption and conformation to inhibit inflammatory cell functions.^24^, ^25^, ^26^, ^27^, ^28^ The objective of this study was to evaluate the effectiveness of this novel scaffold to facilitate new bone formation within segmental bone defects in horses in a minimally invasive model.

We evaluated new bone formation associated with a novel bone filler scaffold placed into bone defects created in the midbody of the fourth metacarpal bones (MCIV) for 60 days. Contralateral limb bone defects were used as internal controls for bone formation and to verify the model. The bone healing model used in this study is well established.^29^, ^30^, ^31^, ^32^, ^33^, ^34^, ^35^ Healing was evaluated by using high‐definition infra‐red thermography, ultrasonography, radiography, computed tomography (CT), and bone histology. We hypothesized that this novel scaffold would improve bone healing and that new bone would form within (in‐growth) as well as on the outer surface (on‐growth) of the scaffolds.

## 2 MATERIALS AND METHODS

### 2.1 Study population and study design

This study was conducted with mares acquired and owned by the institution. The study protocol was approved by the University of Tennessee IACUC (approval No. 2609–0518). Horses were screened for general health and preexisting orthopedic conditions. Physical examination as well as palpation and visual lameness evaluation of the forelimbs while walking in a straight line was performed. Horses with signs of systemic disease (increased body temperature, increased bronchovesicular sounds, nasal discharge, or cough), and forelimb lameness and those with any abnormality associated with the MCIV were excluded from the study. Individual front limb MCIV of the horses were randomly assigned to one of two treatment groups within horse by using a simple randomization with a coin toss such that each MCIV was assigned to either the novel scaffold or control treatment groups. Treatment groups included scaffold (n = 5 MCIV; NuShores Biosciences, Little Rock, Arkansas) or untreated control defect (n = 5 MCIV).

### 2.2 Anesthesia and perioperative management

Perioperative treatment included gentamicin sulfate (100 mg/mL; VetOne, Boise, Idaho; 6.6 mg/kg IV every 24 hours), penicillin procaine G (injectable suspension, 300.000 units/mL; VetOne;, 22 000 IU/kg IM every 12 hours), and firocoxib, a nonsteroidal anti‐inflammatory (Equioxx injectable, 20 mg/mL; Boehringer Ingelheim Animal Health USA, Duluth, Georgia; 0.3 mg/kg IV every 24 hours). The horses were sedated with acetylpromazine maleate (Acepromazine, 10 mg/mL; VetOne; 0.04 mg/kg IM), followed approximately 30 min later by xylazine hydrochloride (Xylamed, 100 mg/mL; VetOne; 1 mg/kg IV). Anesthesia was induced with ketamine hydrochloride (Zetamine, 100 mg/mL; VetOne; 2.2 mg/kg, IV) and midazolam (Akorn, Ann Arbor, Michigan; 0.05 mg/kg IV). Anesthesia was maintained with isoflurane (250 mL; VetOne) in oxygen (3 L/minute) and xylazine hydrochloride (1 mg/kg/hour).

### 2.3 Fourth metacarpal bone segmental ostectomy

Segmental ostectomies of the MCIV were created as previously described.^29^, ^30^, ^31^, ^32^, ^33^, ^34^, ^35^ Briefly, horses were placed in dorsal recumbency, and both forelimbs were clipped from the hoof to above the carpus. The surgical site was aseptically scrubbed and draped accordingly. Length of the MCIV was measured with a sterile ruler from the palpated head of the bone to the palpated distal extremity (button), and the length was divided into three segments. A 5‐cm‐long incision was created over the middle segment of one MCIV. Subcutaneous tissue was bluntly separated to expose the bone, and the periosteum was reflected but left in place. A 2‐cm‐long fragment of the MCIV was removed 1 cm below the proximal margin of the middle segment with an oscillating bone saw (micro oscillating saw; Salvin Dental Specialties, Charlotte, North Carolina). Care was taken not to injure the underlying suspensory ligament and neurovascular bundles during the procedure by retraction from the osteotomy site.

### 2.4 Scaffold preparation and implantation

**FIGURE 1:**
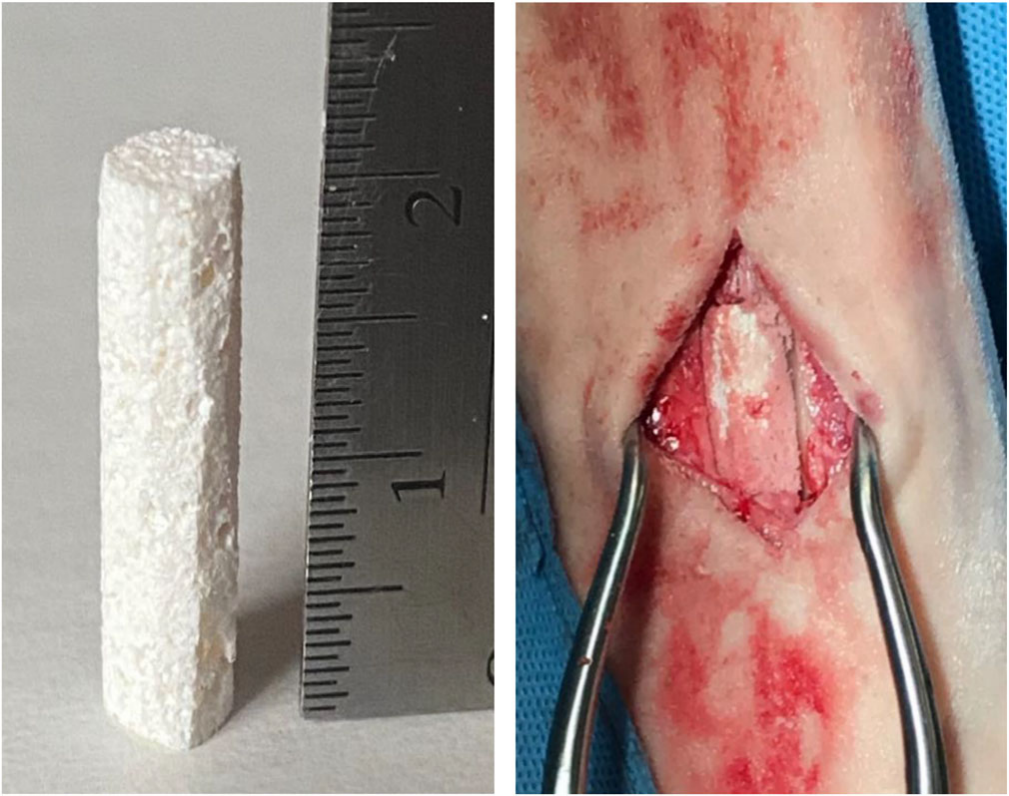
Appearance of the three‐dimensional scaffold. The 2‐cm‐long scaffold before (image at left) and after (image at right) placement in the segmental bone defect created in fourth metacarpal bone. Photographs of bone filler scaffold used with permission of NuShores Biosciences LLC

The bone defect was either filled with scaffold or left unfilled according to treatment group assignment. Prior to implantation within the MCIV defects assigned to the scaffold treatment group, the scaffolds were hydrated with 1 mL of sterile saline. The initial dimensions of the scaffolds were 10 × 10 × 20 mm cubic rectangle. Scaffolds swell slightly when they are hydrated, resulting in a marginal increase in scaffold volume. The scaffold was trimmed by using a scalpel blade to allow the surgeon to precisely fit the dimensions of the defect. The scaffold was malleable, allowing manipulation during the implantation process without damaging its structure (Figure 1). Fourth metacarpal bone defects were left unfilled and served as the within‐subject control comparison group. Subcutaneous tissue was closed in a simple continuous pattern by using absorbable monofilament suture (PDS II polydioxanone 2–0 [3 metric]; Ethicon, Guaynabo, Puerto Rico) with a CT‐1 36‐mm 1/2c taper needle (Ethicon), and skin was closed by using a simple continuous pattern and nonabsorbable monofilament suture (Surgipro polypropylene monofilament 0 [3.5 metric] with a C‐17 39‐mm 3/8 cutting needle; Covidien, Mansfield, Massachusetts).

### 2.5 Postoperative management

Sterile dressings were temporarily placed on both forelimbs, and CT was performed while horses were still under general anesthesia to establish the postoperative characteristics of defects with and without the scaffolds. After CT, sterile distal limb bandages were placed, and the horses were allowed to recover from anesthesia. Antibiotics, penicillin (22 000 IU/kg IM every 12 hours) and gentamicin (6.6 mg/kg IV every 24 hours), were administered for an additional 48 hours. Firocoxib (0.3 mg/kg IV every 24 hours) was administered for an additional 72 hours. Activity of horses was restricted to stall rest for 14 days after surgery, followed by 14 days of turnout into a small pen (4 × 12 m) connected to the stall. Skin sutures were removed 14 days after surgery, and limbs were bandaged for a total of 21 days. After 28 days, horses were turned out to pasture for an additional 30 days.

### 2.6 Postoperative wound assessment

#### 2.6.1 Thermography

Postoperative wound assessment included ultrasonography and thermography^36^, ^37^, ^38^, ^39^, ^40^ to monitor soft tissue swelling, local fluid accumulation, and inflammation. Thermography (Fluke VT04A visual IR thermometer; Fluke Corporation, Everett, Washington) was performed in two projections, anteroposterior and lateral‐medial, maintaining the same distance (0.5 m) from the limb during each recording. The camera was adjusted to the environmental temperature and humidity, and the recording was performed 5 min after bandage removal. Thermography was performed on days 1, 3, 5, 7, 10, 14, 21, 30, and 59 after surgery by one investigator (R.M.G.) who was unblinded.

#### 2.6.2 Ultrasonography

Ultrasonography was performed with a portable ultrasound machine (Logic e Vet NextGEN; Sound Technologies, Carlsbad, California) and a 4.2‐ to 13‐MHz linear transducer (12 L‐RS, Logic e Vet NextGEN; Sound Technologies) in standing horses on days 1, 14, 30, and 59 after surgery. Ultrasonography was performed by one investigator (R.M.G.) who was unblinded. The transducer was placed above the defect parallel to the long axis of MCIV, and longitudinal sections of complete defects were acquired.

### 2.7 Clinical assessment of bone healing

#### 2.7.1 Radiography

Dorsolateral‐palmaromedial 45° oblique projection of each MCIV was performed by using a portable radiograph system (NEXT Equine DR; Sound Technologies) in standing horses on days 1 (Figure S1), 14, 30, and 59 after surgery. The radiographs were taken by one investigator (R.M.G.). The x‐ray generator was positioned 1 m away from the limb, parallel to the ground, and 45° away from the sagittal axis of the limb. The radiographs were focused on the middle of MCIV. The exposure settings, 60 kV and 0.08 mA, were consistent for all radiographs.

**FIGURE 2:**
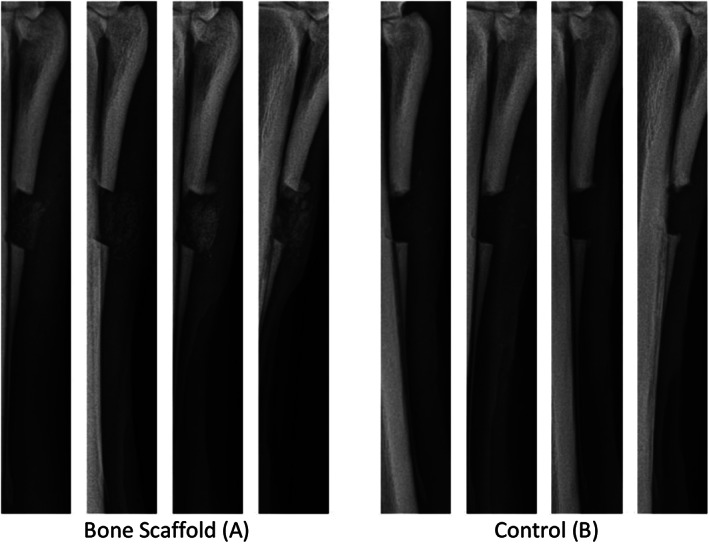
Representative sequence of radiographs at (starting from the left) 24 hours and 14, 30, and 60 days after implantation scaffold within defect (A) and untreated control (B). Defects were created in the fourth metacarpal bones of the same horse. Gradual filling of defects containing scaffold with new bone (A) compared with control (B) in which bone regeneration was minimal

#### 2.7.2 Computed tomography

**FIGURE 3:**
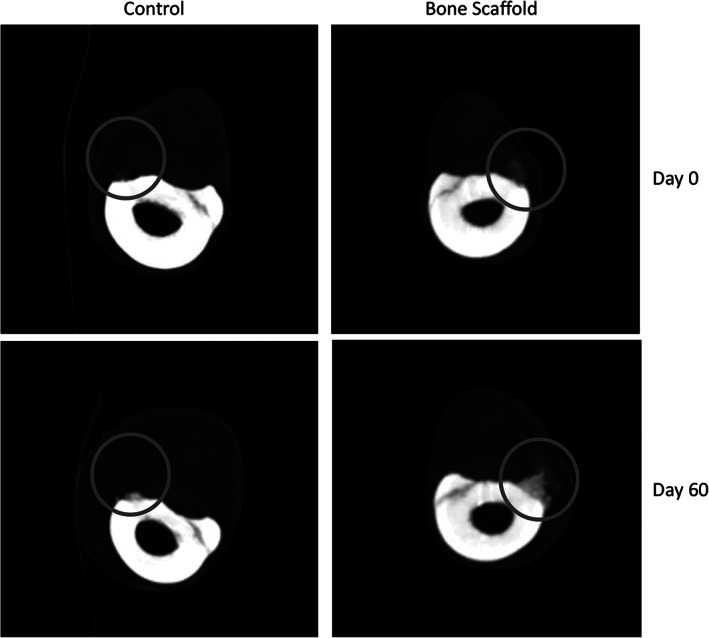
Transverse reconstructed computed tomographic image slabs spanning the length of MCIV defects at days 0 and 60. The defects in MCIV can be seen (gray circles). For control defect, there was a small amount of new bone within the defect at 60 days (density, 143 HU at 0 days vs 429 HU at 60 days; difference, 286 HU). For the scaffold‐treated defect, there was an increase in density of the graft as well as mild peripheral new bone formation at 60 days (density, 403 HU at 0 days vs 826 HU at 60 days; difference, 423 HU). HU, Hounsfield units; MCIV, fourth metacarpal bones

Computed tomography was performed (40‐slice helical CT, Philips Brilliance‐40; Philips International BV, Amsterdam, Netherlands) immediately after surgery (baseline) and again 60 days postimplantation immediately before tissue biopsy (Figure 3). Prior to imaging, CT was calibrated according to manufacturer specifications. Transverse images were reconstructed in 0.67‐ and 5‐mm slice thickness, and sagittal and dorsal multiplanar reconstructed images were generated. Images were uploaded into a digital archive (Sectra PACS IDS7; Sectra AB, Linkoeping, Sweden).

### 2.8 Tissue biopsy and histology

#### 2.8.1 Biopsy

At the termination of the study period (60 days postimplantation), horses underwent CT and tissue biopsy. Perioperative treatment and general anesthesia were administered as previously described. Computed tomography of both forelimbs was performed, and forelimbs were prepared for aseptic surgery. A 5‐cm‐long incision was created over the defect in both forelimbs. Subcutaneous tissue was bluntly separated to expose the defect, and a 5‐mm‐long, transverse segmental biopsy was performed at the center of the defect by using a scalpel blade or osteotome when required. Subcutaneous tissue and skin were closed as previously described. Sterile distal limb bandages were applied to both forelimbs, and the horse was allowed to recover. Tissue was submitted for histological evaluation. Postoperative care was administered as previously described.

#### 2.8.2 Histology

Histology was performed on decalcified specimens. Each tissue specimen was preserved in 10% neutral buffered formalin for 48 hours after biopsy and was then decalcified with 15% formic acid for up to 14 days after tissue harvest. Specimens were sectioned and stained with hematoxylin and eosin and Masson's trichrome for histologic evaluation. Histologic sections were analyzed by a board‐certified pathologist (R.L.D., who was blinded) using a light microscope at ×10, ×20, and ×100.

### 2.9 Data generation from assessment methods

Collected data were organized in Excel 2016 (Microsoft, Redmond, Washington). Thermography data included average Celsius temperature of the skin surface from both treatment groups that was measured at each time point. The images recorded during ultrasonography were recorded and subjectively described by one investigator (R.M.G.).

Radiographs and CT images were qualitatively evaluated by a board‐certified radiologist (S.H., who was blinded to the treatment group) and scored for ostectomy gap filling and opacity of new bone (Tables S1, S2 and S3). The individual ostectomy gap filling and opacity scores were summarized for an overall radiographic healing score for each defect. Quantitative assessment of defect filling with new bone was performed on radiographic images in ImageJ (NIH Fiji package; National Institutes of Health, Bethesda, Maryland). The images underwent 8‐bit transformation, and binary masks were created by adjusting the grayscale threshold to highlight bone. Quantitation was conducted over a previously drawn region of interest (ROI) covering the area of the defect. Defect filling was calculated and expressed as percentage of the area of the defect.

Computed tomographic images were scored for new bone formation by the radiologist (S.H.), who was blinded. A transverse slab was reconstructed spanning the length of the defect for both the study limb and the control limb, and the density of the defect was measured quantitatively (Hounsfield units [HU]).

Quantitative assessment of bone formation was performed by using histologic slides stained with Masson's trichrome in Image J (NIH Fiji package; National Institutes of Health). Digital images (×2) of the histological sections were taken. The binary masks were created by adjusting color threshold to highlight only bone (dark intense blue color) and by drawing the ROI covering the entire cross‐section of the biopsied defect. Five images across the scaffold were analyzed, and the average bone formation was calculated and expressed as percentage of tissue cross‐sectional area.

### 2.10 Statistical analysis

Statistical analysis was performed in SAS 9.4 TS1M6 (SAS Institute, Cary, North Carolina) and in PS Power and Sample Size Calculations (ver 3.0, 2009; https://vbiostatps.app.vumc.org/ps/). Descriptive statistics were determined for the continuous variables including thermography, percentage of defect filling, and CT measurement of defect density. The continuous variables, including percentage of defect filling with bone and CT measurements of defect density immediately after surgery and before biopsy as well as the ordinal variables including new bone score, opacity score, and ostectomy gap filling score were analyzed by using mixed‐model analysis for randomized block design with the individual animal as blocked effect. Thermography data were analyzed by using repeated‐measures analysis of variance. Normality of data distribution and equality of variance were evaluated with Shapiro–Wilk and Levene's tests, respectively. Ranked transformation was applied when diagnostic analysis of residuals violated the assumptions of normality of data distribution and equality of variance. Post hoc multiple comparisons were performed with Tukey's adjustment. Statistical significance was assumed at α = .05. The calculated power of the study design and carried out evaluations to detect the true difference in the filling of the defect with the new bone were β = .9.

## 3 RESULTS

The study included five adult mares, 4 to 10 years old and weighing 437.7 kg ± 29 kg. The study population included one each of thoroughbred, quarter horse, Rocky Mountain horse, standardbred, and mixed‐breed horse. No adverse effects were detected after implantation in any horse at either surgical site. However, slight proximal displacement of the scaffold occurred in one horse within 14 days of surgery. Design of the study, in which each horse had a scaffold‐treated defect and an untreated defect, precluded true blinding of investigators because of the obvious nature of the treatment. In an effort to minimize bias, individual investigators were assigned to analysis of each data set without knowledge of the others' interpretation.

### 3.1 Postoperative wound assessment

According to thermography analysis, skin surface temperatures were similar at all times after surgery for both surgery sites in each horse (*P* > .05). The skin surface temperature between 7 and 10 days after the surgery was 35.22°C ± 0.8°C in scaffold‐treated defects and 35.39°C ± 1.1°C in control‐treated defects (*P* > .05); skin surface temperature was lower for the remaining 50 days (*P* < .05) by 2.18°C ± 1°C in scaffold and by 2.10°C ± 0.7°C in control group (*P* > .05).

Ultrasonography findings were consistent with increasing opacity as a result of new bone formation on the surface of the scaffolds. This was evident as smooth hyperechogenic lines on the surface of the implant (Figure S1). The defect on the control side remained unfilled, which was evident as a hypoechogenic area within the defect and clear delineation of the proximal and distal bone margins. Fourteen days after implantation, mild to moderate homogeneous fluid accumulation was found at both surgery sites in each MCIV. The fluid accumulation was no longer present 30 days after surgery.

### 3.2 Clinical assessment of bone healing

#### 3.2.1 Radiography

Sixty days after surgery, MCIV defects treated with scaffolds had greater bone filling (67.42% ± 26.7%) compared with control defects (35.88% ± 32.7%; *P* = .006; Figure 2). One horse had limited bone response affecting both bone defects during the study period. In this horse, the defect treated with a scaffold had 25.49% defect filling, and the control defect had 1.51% defect filling. Mild to moderate periosteal reaction was observed at the distal ends of proximal bone segments in defects treated with a scaffold. This reaction was also present in control defects, in which minimal bone formation was noticed. On the basis of qualitative gap filling scores of the radiographs, defects treated with the scaffold had a greater median score (median, 2) compared with control defects (median, 1; *P* = .033). Defect opacity was not different between the treatment groups (*P* = .070).

#### 3.2.2 Computed tomography

The average density of the defects treated with scaffolds immediately after surgery was 449.8 ± 137.1 HU, and that of the unfilled defects was 83.20 ± 133.4 HU (*P* = .003). The density was increased throughout the study by 441.20 ± 128.3 HU in the defects treated with scaffolds (*P* = .003) and by 381.60 ± 198.4 HU in the unfilled defects (*P* = .001). Sixty days after implantation, the density of the defects treated with scaffold was 807.80 ± 129.6 HU, and that of the unfilled defects was 464.80 ± 81.3 HU (*P* = .004; Figure 3). The HU range for trabecular bone has been reported as between 136 and 507.6 HU in the literature.^41^ Qualitative scoring of CT images confirmed greater bone formation within scaffold‐treated defects (median, 2/4) compared with the controls (median, ¼; *P* = .001).

#### 3.2.3 Histology

**FIGURE 4:**
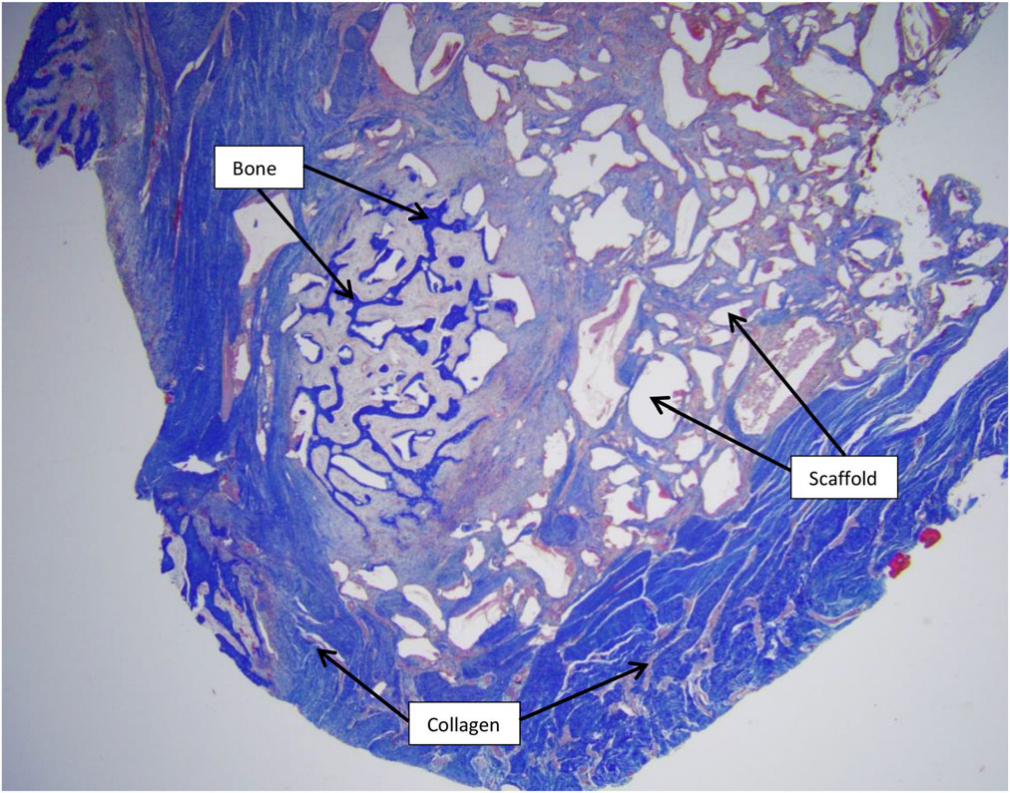
Histological appearance of the central aspect of defects filled with the scaffold; bone formation can be seen within the pore channels of the scaffold as well as spaces representing the scaffold (white areas). The collagen surrounding the implant on its outer surface is consistent with good integration of the implant. Masson's trichrome stain; ×2

**FIGURE 5:**
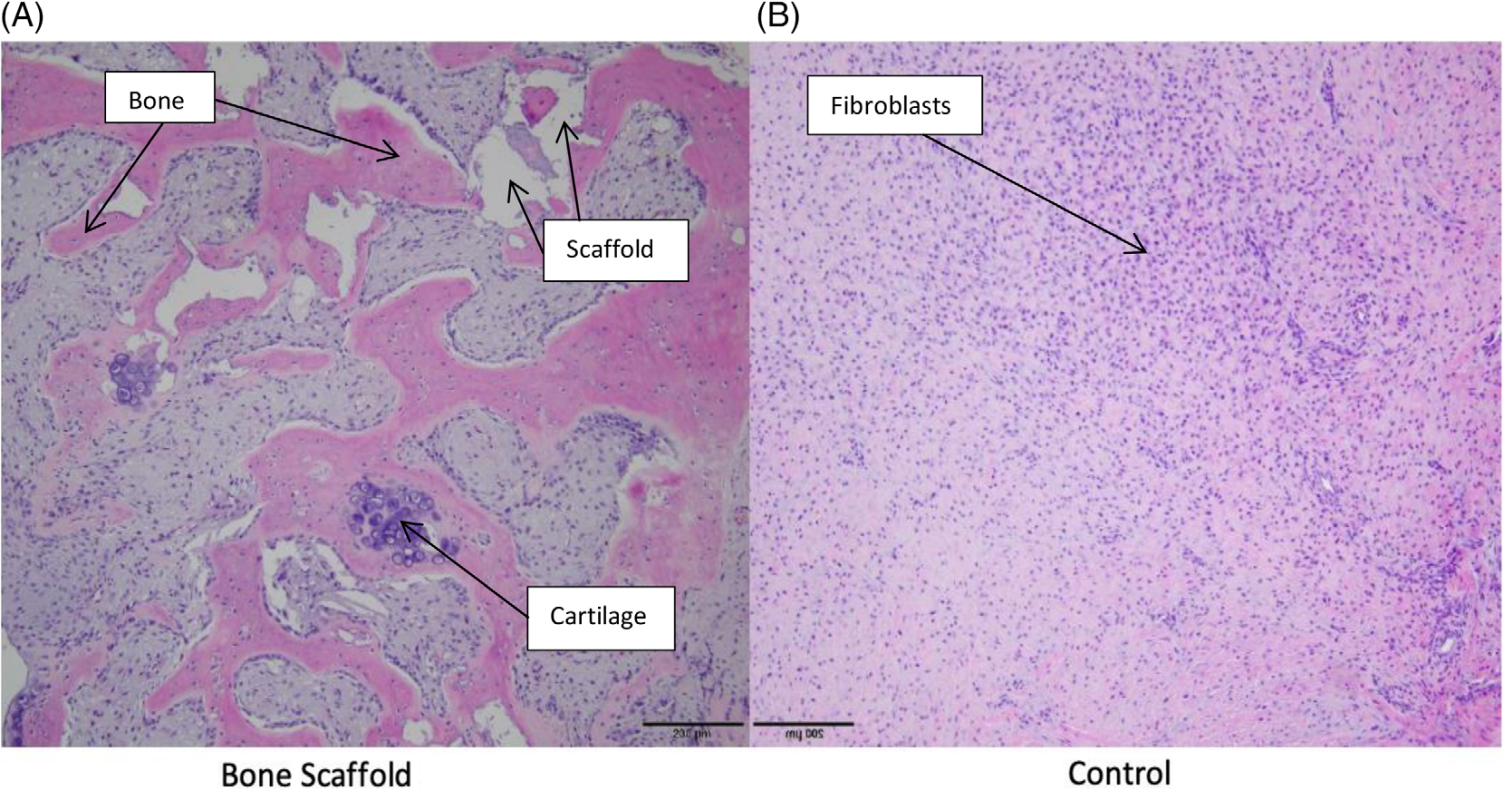
Histological appearance of the central aspect of defects filled with the regenerative scaffold (A) and control (B) from the same horse; bone formation can be seen within the pores of the scaffold as well as spaces filled with polyurethane masses (white areas). A small island of cartilage was found in this horse. The tissue harvested from middle of the control defect represents connective tissue without signs of new bone formation. Hematoxylin and eosin stain; ×10

New bone formation was found within (in‐growth) as well as on the surface (on‐growth) of the scaffolds (Figure 4). New bone tissue was formed within an average of 9.43% ± 3.7% of scaffold cross‐sectional area. In one horse, a small island of hyaline cartilage formation was identified (Figure 5). The remaining area of the implant cross‐section was occupied by mixtures of new bone and scaffold material. In contrast, the control biopsies were predominantly filled with fibroblasts and collagen with no signs of bone formation (Figure 5). Quantitation of bone tissue formation was therefore not possible in controls.

## 4 DISCUSSION

In this study, the novel scaffold supported healing of experimental segmental defects created in the splint bones of normal horses within 60 days. The study used a splint bone segmental defect model that has been previously established as an acceptable model for evaluating equine bone healing with minimal animal morbidity and that is minimally invasive.^16^, ^29^, ^30^, ^31^, ^32^, ^33^, ^34^, ^35^ The use of a bilateral splint bone defect model enabled a smaller number of horses to be used and reduced variability when comparing treatment response.

Healing within the defects treated with scaffolds was compared with healing of the empty defects in the contralateral limbs that served as the within‐subject control. The bone defects created in MCIV in this study were larger than those previously reported in the literature. Previous studies have used smaller defects, ranging from 0.1 to 1 cm, with inconsistent results.^16^, ^29^, ^30^, ^31^, ^32^, ^33^, ^34^, ^35^ The rationale for the larger size of the defect in the study reported here was to create a critical‐sized defect. By definition, a critical‐sized defect will not heal spontaneously during the lifetime of the animal.^42^, ^43^, ^44^ Segmental bone defects have been further defined as having a length exceeding 2 to 2.5 times the diameter of the affected bone.^42^, ^43^, ^44^ Presence of the empty defect was required to confirm that defects created in MCIV achieved a critical size for the purposes of this 60‐day study. The defect size of 2 cm satisfied the requirements of the study because none of the negative controls healed within the study period. Bone formation was noticed only in the defects treated with scaffolds.

In this study, the novel scaffold supported early bone formation. The amount of newly formed bone varied similarly within the treatment groups. One horse presented less bone formation within both defects, providing evidence that this horse may have had an individual physiologic influence that limited bone healing. The data provide evidence to support our hypothesis that this novel scaffold can be used to support bone formation in horses and that the implant is osseointegrated over time. Data from a previous in vitro study in which researchers used PU platforms embedded with nanophase HA and DBP provided sufficient evidence to conclude that this platform is cytocompatable and supports osteoblastic proliferation.^22^ Results of an in vivo study in rats in which researchers used a tibia model provided evidence that this scaffold is biocompatible and supports new bone formation to aid in healing of the created defect.^44^ Results of the current study provide evidence that this novel scaffold platform supports bone regeneration in horses, including a larger defect than previously described.^16^, ^31^, ^32^, ^33^, ^34^, ^35^ Furthermore, in contrast to previously published studies in which researchers compared the treatment groups to the negative controls, in the study reported here, we describe a greater difference between the treatment group and the untreated controls in the filling of the defects as well as measured tissue density within the defects.^16^, ^31^, ^32^, ^33^, ^34^, ^35^

This novel scaffold supported bone formation on its outer surface (on‐growth) and within the implant (in‐growth) without formation of a fibrous tissue interlayer between the implant‐bone surfaces, providing evidence of successful osseointegration (Figure 5).^15^ An island of hyaline cartilage formed within the defect in one horse, but the cause of this formation is unknown. The cartilage formation could have been related to the local tissue environmental factors during early bone regeneration.^45^ Mild to moderate periosteal reaction was present on the distal margins of proximal bone segments in all defects treated with the scaffold. This reaction was much less pronounced in untreated defects and can be attributed to the splint bone defect healing supported by the scaffold.

Despite the small treatment group, the study design yielded statistically significant results and achieved sufficient power (study power, β = .9) for data interpretation. Scaffold manipulation as well as implantation was feasible and was completed in all cases. Although one scaffold became mildly displaced from the defect in a proximal and abaxial direction, this displacement did not interfere with the bone response, and no untoward effects were observed.

According to computed tomographic images, the defects filled with novel scaffolds had greater density than the defects in the control treatment group. The scaffold architecture contains bone mineral particles, which might have contributed to the initial density of the defects immediately after the surgery. However, density increased within the defects treated with scaffolds and was consistent with new bone formation. This was also supported by results of the histological evaluation, which provided evidence to confirm bone formation within the scaffolds as well as on the surface of the implant. Direct comparison of the density changes between the defects containing scaffolds and the control defects is challenging because of the composition of the implant. It is unknown to what extend the density measured at 60 days was affected by the presence of bone mineral particles and the PU masses and how much was affected by the newly formed bone.

Few synthetic scaffolds such as CMHA‐SGX sponge, collagen‐HA scaffold, multilayer scaffold containing collagen types I and II, hyaluronic acid, HA, or bi‐layered scaffold consisting of polyetherketoneketone and PU elastomer have been studied in horses.^16^, ^17^, ^18^, ^19^ The features of the novel scaffold used in this study are unique compared with other synthetic scaffolds^21^, ^22^ mainly because of the scaffold's hydrophilic nature, which allows for filling of nonuniform bone voids and small incongruences. The combination of soft (PU) and hard (HA and DBP) components gives an overall mechanical integrity greater than that of the individual components.^28^ The novel scaffolds are implanted into bone defects in a press‐fit manner. The scaffold is designed to have a wide range of porosities intended to support neovascularization, cell migration, and bone formation.^21^ Variable porosity, ranging from 150 to 800 μm, has been reported to be ideal for new bone formation because small pores (<150 μm) facilitate early cellular migration with microvascularization, and large pores (>300 μm) facilitate larger blood vessel formation, resulting in vascularization of the graft and sustained tissue formation.^46^, ^47^, ^48^

This technology was tested in the equine splint bone model, which is a well‐established model to research bone healing in horses.^29^, ^30^, ^31^, ^32^, ^33^, ^34^, ^35^ The splint bone segmental ostectomy is considered a safe method, with minimal soft tissue disruption, that does not cause exostosis or sequestration formation.^29^ Furthermore, this procedure does not impair perfusion or integrity of the distal segment, which likely is maintained from the support of periosteal and soft tissue vasculature.^29^ Horses tolerated the procedure well with a lack of noticeable pain, discomfort, or gait impairment monitored by visual lameness assessment. The interosseous ligament coursing between the fourth and third metacarpal bones provides a relatively stable ostectomy model, with the advantage of not requiring internal fixation.^29^, ^30^, ^31^, ^32^, ^33^, ^34^, ^35^ Each horse can serve as its own control by using splint bones in other limbs and thereby controlling for intraindividual factors to limit variability (eg, paired data) as opposed to interindividual variability, which has been seen in other models.^32^ The limitation of this model is related to the fact that the fourth and second metacarpal bones are not fully weight‐bearing bones and do not contain a medullary cavity. Therefore, the entire healing process occurs from the periosteum and recruitment of cells from the surrounding tissues.

Our study design had several limitations. Wound healing evaluation with thermography and ultrasonography were performed unblinded, and bone healing evaluation with radiography, CT, and histology were performed blinded. The radiologist was, however, aware of the timeline of the radiographic images. Infrared thermography also has limitations. This method uses infrared waves that are reflected from the skin surface and superficial tissues. It measures surface temperature that has been correlated with blood flow. This method has used in several studies as an ancillary diagnostic to evaluate joint diseases; laminitis; and injuries of the long bones, tendons, ligaments, muscles, and vertebral column in horses.^36^, ^37^, ^38^, ^39^, ^40^ Because of the large number of factors that influence the quality of the thermogram, including factors related to the animal, environment, and acquisition, the outcome should be interpreted with caution.^38^, ^39^ Finally, the histology sections were harvested from the center of the defects, and the ends of MCIV were not included in the analysis. This precludes definitive interpretation of the defect bone and scaffold/bone interfaces.

The novel scaffold described in the study reported here supported splint bone regeneration during the 60‐day period after implantation. Results were consistent among horses, with relatively small standard deviation between the individual horses. This technology offers a viable bone filler for use in bone voids in horses. Future research directions may include comparison of the scaffold to autologous bone grafts to determine whether there is equivalent or improved efficacy compared with other established methods of bone regeneration.

## AUTHOR CONTRIBUTIONS

Grzeskowiak RM, DVM, PhD: Performed surgeries and diagnostic imaging, acquired, organized, and analyzed data, wrote the manuscript, and read and approved the final submitted manuscript; Alghazali KM, MS, PhD: Constructed the scaffolds, analyzed data, wrote the manuscript, and read and approved the final submitted manuscript; Hecht S, DMV, DACVR, DECVDI: Performed computed tomography and diagnostic imaging evaluation, edited the manuscript, and read and approved the final submitted manuscript; Donnell RL, DVM, PhD: Performed histologic evaluation, edited the manuscript, and read and approved the final submitted manuscript; Doherty TJ, DVM, DACVAA: Performed general anesthesia and edited the manuscript; Smith CK, DVM, DACVAA: Performed general anesthesia, edited the manuscript, and read and approved the final submitted manuscript; Anderson DE, DVM, MS, DACVS: Supervised the project, performed data acquisition and analysis, edited the manuscript, and read and approved the final submitted manuscript; Biris AS, MS, PhD: Supervised the project, performed scaffold construction, wrote the manuscript, and read and approved the final submitted manuscript; Adair HS, DVM, MS, DACVS, DACVSMR: Supervised the project, performed surgeries, diagnostic imaging, data acquisition, and analysis, wrote the manuscript, and read and approved the final submitted manuscript.

## CONFLICT OF INTEREST

D.E. Anderson and A. S. Biris A.S. have co‐ownership in the regenerative novel scaffold technology used in this research that has been licensed by the University of Arkansas at Little Rock to NuShores Biosciences LLC. All other authors declare no conflicts of interest related to this report.

## SUPPORTING INFORMATION

Additional supporting information may be found online in the Supporting Information section at the end of this article.

## Supporting information

**Figure S1** Representative sequence of ultrasonography images recorded throughout the study of defect filled with scaffold (A) and untreated control (B). Both defects were created in the same horse. Each sequence consists of four images which were recorded at 24 h (starting from top to bottom), 14, 30 and 60 days after the implantation surgery. The images show increased soft tissue swelling as well as fluid accumulation 14 days after implantation (second row of images from the top) that was similar in both treatment groups. The bone scaffold is increasingly covered with bone presented as the smooth hyperechogenic line (A). In the control defect, bone formation was minimal and occurred only at the margins of the defect (B).

**Appendix** S1: Supporting Information
**Table S1.** Radiographical ostectomy gap filling scoring system.
**Table S2.** Radiographic opacity score.
**Table S3.** CT ostectomy gap filling scoring system.

**Figure S2.** Radiographs obtained 240 days after defect creation and 180 days after the termination of the study: scaffold (A) and the control (B). Both defects were created in the same horse. The scaffold (A) is degraded and incorporated within the new bone.
