# Supplementary material for: Influence of a novel scaffold composed of polyurethane, hydroxyapatite, and decellularized bone particles on the healing of fourth metacarpal defects in mares
Source: Vet Surg. 2021 May 5;50(5):1117–27. doi: 10.1111/vsu.13608 (PMC8360067; doi:10.1111/vsu.13608)
Supplement: Supplementary file 1 — Figure S1 Representative sequence of ultrasonography images recorded throughout the study of defect filled with scaffold (A) and untreated control (B). Both defects were created in the same horse. Each sequence consists of four images which were recorded at 24 h (starting from top to bottom), 14, 30 and 60 days after the implantation surgery. The images show increased soft tissue swelling as well as fluid accumulation 14 days after implantation (second row of images from the top) that was similar in both treatment groups. The bone scaffold is increasingly covered with bone presented as the smooth hyperechogenic line (A). In the control defect, bone formation was minimal and occurred only at the margins of the defect (B). [file VSU-50-1117-s002.pdf]

Supplementary figure.

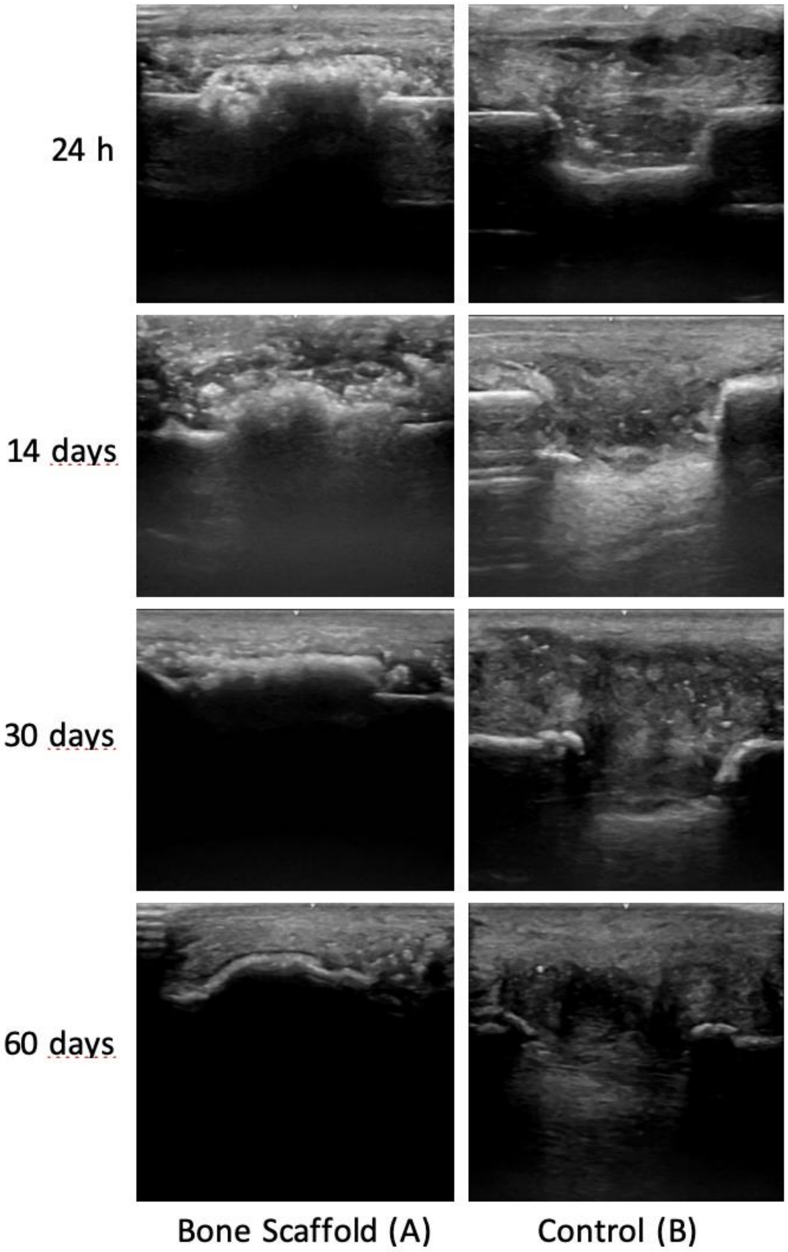

Representative sequence of ultrasonography images recorded throughout the study of defect filled with scaffold (A) and untreated control (B). Both defects were created in the same horse. Each sequence consists of four images which were recorded at 24h (starting from top to bottom),

14, 30 and 60 days after the implantation surgery. The images show increased soft tissue swelling as well as fluid accumulation 14 days after implantation (second row of images from the top) that was similar in both treatment groups. The bone scaffold is increasingly covered with bone presented as the smooth hyperechogenic line (A). In the control defect, bone formation was minimal and occurred only at the margins of the defect (B).
