# Supplementary material for: Influence of a novel scaffold composed of polyurethane, hydroxyapatite, and decellularized bone particles on the healing of fourth metacarpal defects in mares
Source: Vet Surg. 2021 May 5;50(5):1117–27. doi: 10.1111/vsu.13608 (PMC8360067; doi:10.1111/vsu.13608)
Supplement: Supplementary file 2 — Appendix S1: Supporting Information Table S1. Radiographical ostectomy gap filling scoring system. Table S2. Radiographic opacity score. Table S3. CT ostectomy gap filling scoring system. [file VSU-50-1117-s001.pdf]

Supplementary Tables

Table 1. Radiographical ostectomy gap filling scoring system.

Ostectomy gap filling	Score
**No interval change compared to immediate post-operative radiographs**	0
**New bone filling <25% of ostectomy gap**	1
**New bone filling 26-50% of ostectomy gap**	2
**New bone filling 51-99% of ostectomy gap**	3
**Ostectomy gap completely filled and/or bridging callus present on at least 3 cortices**	4

Table 2. Radiographic opacity score.

Opacity of new bone	Score
**No interval change compared to immediate post-operative radiographs**	0
**New bone less opaque than normal cortex**	1
**New bone of similar or greater opacity compared to normal cortex**	2

Table 3. CT ostectomy gap filling scoring system.

CT findings	Score
**No evidence of new bone formation adjacent to graft or within ostectomy gap**	0
**New bone filling <25% of ostectomy gap**	1
**New bone filling 26-50% of ostectomy gap**	2
**New bone filling 51-99% of ostectomy gap**	3
**Ostectomy gap completely filled and/or bridging callus present on all cortices**	4
