# Supplementary material for: Influence of a novel scaffold composed of polyurethane, hydroxyapatite, and decellularized bone particles on the healing of fourth metacarpal defects in mares
Source: Vet Surg. 2021 May 5;50(5):1117–27. doi: 10.1111/vsu.13608 (PMC8360067; doi:10.1111/vsu.13608)
Supplement: Supplementary file 3 — Figure S2. Radiographs obtained 240 days after defect creation and 180 days after the termination of the study: scaffold (A) and the control (B). Both defects were created in the same horse. The scaffold (A) is degraded and incorporated within the new bone. [file VSU-50-1117-s003.docx]

Figure S2


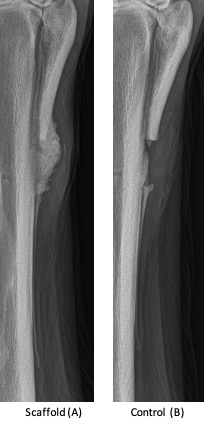


*Figure S2* Radiographs obtained 240 days after defect creation and 180 days after the termination of the study: scaffold (A) and the control (B). Both defects were created in the same horse. The scaffold (A) is degraded and incorporated within the new bone.
